# Prevalence of Radix Entomolaris in Mandibular Permanent Molars Analyzed by Cone-Beam CT in the Saudi Population of Ha'il Province

**DOI:** 10.7759/cureus.47034

**Published:** 2023-10-14

**Authors:** Moazzy I Almansour, Ahmed A Madfa, Adhwaa F Algharbi, Reem Almuslumani, Noeer K Alshammari, Ghufran M Al Hussain

**Affiliations:** 1 Restorative Dental Science, College of Dentistry, University of Ha'il, Ha'il, SAU; 2 Dentistry, Alghassab Dental Clinic, Ha'il, SAU; 3 Dentistry, Alanwar Hospital, Ha'il, SAU; 4 Dentistry, Sakha Medina Hospital, Ha'il, SAU

**Keywords:** anatomy, saudi subpopulation, cone beam computed tomography, radix entomolaris, mandibular molars

## Abstract

Background: The present study aimed to examine the prevalence of radix entomolaris (RE) in the mandibular permanent molar within a specific sub-population in Saudi Arabia.

Methods: A comprehensive analysis was conducted on 499 cone-beam computed tomography (CBCT) scans of a mandibular molar from a sample of Saudi patients aged between 18 and 65. The primary objective of this study was to investigate the anatomical characteristics of mandibular permanent molars, specifically focusing on the number of roots present. The chi-square test was employed to examine the relationship between various variables.

Results: In the case of mandibular first molars, it was observed that 95.3% of these molars exhibited a bifurcated root structure. In comparison, the remaining 4.7% displayed a triradicular configuration within the sample population under investigation. Although there were some variations, no significant differences in the number of roots were observed between males and females or left and right sides (p > 0.05). In the case of mandibular second molars, it was observed that 96.9% of them exhibited a bifurcated root structure, whereas 2.5% displayed a trifurcated root configuration, and a mere 0.6% possessed a single root. There were no statistically significant variations in the number of roots between males and females or left and right sides (p > 0.05).

Conclusions: The identification of RE was observed in the mandibular molars. Moreover, the discovered RE roots were predominantly found in the mandibular first molar, displaying a tendency for bilateral occurrence in both male and female individuals.

## Introduction

A comprehensive understanding of the anatomical modifications associated with the root canal system is crucial for the effective execution of root canal therapy (RCT). Insufficient eradication of germs and the prolonged presence of inflamed or infected pulp tissue might result from a lack of precise identification and treatment of anatomical and morphological irregularities. Consequently, this might lead to the occurrence or continuation of apical periodontitis (AP). Several studies have provided evidence that indicates a higher prevalence of AP in teeth that have not received root canal treatment [1,2]. Hence, gaining a thorough understanding of anatomical structures allows for the anticipation of probable challenges that may occur during and after RCT, thereby improving the effectiveness of the treatment.

The complicated configuration of mandibular permanent molars poses significant challenges when it comes to their proper cleaning and shaping. According to previous scholarly works, it has been observed that the mesial root of mandibular molars commonly exhibits two canals, but the distal root may possess either one or two canals [3]. In general, the mandibular first and second molars exhibit mesial and distal root orientations, with each root possessing a total of three canals [4]. However, it is important to acknowledge that in some individuals, there may be an occasional occurrence of a third root due to ethnic disparities or variations in tooth form, particularly in the lingual region [5,6]. The nomenclature 'radix entomolaris' (RE) was designated by Bolk in 1915 [7]. When compared to other root structures, RE demonstrates a proclivity for shorter lengths and a coiled arrangement, enabling their potential connection or separation from roots [8,9]. Carlsen and Alexandersen (1990) classified the morphology of RE [10], whereas Ribeiro and Consolaro (1997) investigated their buccolingual orientation [8].

The frequent observation of an additional distolingual root (DLR) and distolingual canal (DLC) has been documented [11]. Another morphological difference that can be detected in dental anatomy is referred to as the RE. This particular type refers to the presence of a third root positioned distolingually, which is commonly characterized by its reduced length and more prominent curvature when compared to the distobuccal (DB) root. The presence of a canal located at the root end, often known as the root end canal, presents challenges in terms of its morphology, debridement process, and obturation technique when compared to the main root canal, referred to as the DB canal [12]. The DLC is located at a greater distance from the major distal canal, necessitating modifications to the conventional access cavity. This modification involves the fabrication of a trapezoidal structure to accurately ascertain the entry point to the DLC [13].

The DLRs of the mandibular molars are not simply an elongation of the distal root. Instead, they are a separate root structure that has its own discrete root canal opening and a unique apex [14]. They display a variety of morphological features, including a truncated conical protrusion or a completely grown root with a standard length and a root canal [14]. Previous research has demonstrated that the DLRs of mandibular molars generally have smaller diameters and exhibit higher curvature in comparison to other types of roots. Moreover, it has been observed that these deep learning roots exhibit a greater tendency towards the buccal direction in their apical area, as indicated by previous studies [15-17]. The identification of a C-shaped canal is a frequently seen anatomical variation in the mandibular second molar within the East Asian population [18]. The coexistence of DLRs in the mandibular first molar and C-shaped canals in the mandibular second molar, which are notable morphological variations reported in individuals of East Asian descent, has not been comprehensively examined in the current body of scholarly research.

To ensure the effective completion of RCT, it is important to acquire a thorough comprehension of the complex internal architecture of the root canal system and its diverse irregularities [19]. The application of preoperative radiographic imaging can reveal substantial data regarding the shape of root canals, encompassing the exact number of roots and canals. This enables healthcare professionals to make informed decisions regarding the selection of the most advantageous approach for endodontic treatment, thereby enhancing the probability of achieving a favorable outcome [4,5]. The mandibular first molars frequently exhibit a canal layout consisting of three to four canals and are typically distinguished by the presence of two roots. Nevertheless, it is important to note that variations in this anatomical structure can be observed. The term 'radix molaris' (RM) refers to the occurrence of an extra root in the mandibular first molar. The occurrence of the supernumerary root is frequently observed in a distolingual position, with a prevalence rate ranging from 0.7% to 33.1%, according to various studies [6-9,20-23].

Numerous studies have shown the prevalence of RE in mandibular molars across various nations. However, it is crucial to conduct a focused investigation on the occurrence of this pattern within the population of Saudi Arabia. Therefore, this study aimed to investigate the occurrence of RE in the mandibular permanent molars among a particular subgroup of individuals in Saudi Arabia.

## Materials and methods

A retrospective cross-sectional observational approach was employed in a study conducted in the Ha'il area of Saudi Arabia to investigate the root and canal morphology of mandibular molars. The Medical Ethics Committee of the University of Ha'il formally approved this study (No. H-2021-025). Images obtained utilizing cone-beam computed tomography (CBCT) technology for diagnostic purposes from May 2020 to November 2022 were included in the study. Given the retrospective nature of the study, it was exempted from informed consent by the ethical council of the College of Dentistry. Throughout the duration of this investigation, the investigators documented the gender and bilateral similarity of the participants. To ensure the preservation of patient confidentiality, the material was handled in a manner that upheld its confidential nature.

The study employed purposive nonprobability sampling to examine a dataset of 2000 CBCT images. Only those CBCT scans presenting distinct visual representations of mandibular permanent molars and robust root structures that are characteristic of persons within the age range of 18 to 65 years were included in the study. The present study did not include photos that portrayed teeth with full-coverage or metallic restorations, endodontic or post-coronal treatment, or scan artifacts. Furthermore, teeth that displayed periapical problems, root resorption, calcification, and inadequate CBCT image quality were eliminated from the research.

The determination of the sample size was conducted utilizing Cochran's formula for estimating sample size: N = (Zα ×P (1-P))/D^2. The recommended sample size for this investigation was 245 dental specimens. Following a comprehensive assessment of 2000 pictures using predetermined criteria for inclusion and exclusion, the resultant sample size for the study consisted of 250 CBCT scans. The final sample comprised a total of 499 teeth.

The Carestream CS 8100 3D imaging equipment (Carestream Dent LLC, Atlanta, USA) was utilized per the recommended methodology of the manufacturer. The images were evaluated using the CS 3D imaging software (Internal Version 3.10.8.0, Carestream Dent LLC, Atlanta, USA). To enhance the fidelity of the depiction, the software's picture editing capabilities were employed to adjust the sharpness, brightness, and contrast of the photographs. The investigation on the number of roots present in mandibular molars was carried out separately at three specific orientations, specifically the axial, coronal, and sagittal planes.

Before carrying out the assessment, the examiner participated in calibration training. The examiners were calibrated under the supervision of authors A.A.M. and M.I.A. in accordance with specified norms and variations before completing the experimental reading. The examiners utilized a random selection technique to investigate a subset comprising 20% of the sample. The degree of concordance among observers was subsequently evaluated by calculating the kappa coefficient, resulting in a value of 0.89. The participants actively participated in a collective endeavor to assess and deliberate upon situations of disagreement, ultimately reaching a final agreement. After the initial assessment, the examiner proceeded to perform a further analysis while retaining impartiality. Approximately 20% of the sample was used for this purpose, aiming to assess the consistency of observations made by the same examiner. The degree of consensus among observers, as quantified by the measure of intra-observer agreement, was found to be 0.92.

Data was analyzed using SPSS Statistics (IBM Corp., Armonk, NY, USA). This software facilitated several statistical procedures, such as frequency distribution and cross-tabulation. The analysis examined the organization of roots. The chi-square and Fisher's exact tests were employed to assess the potential association between the gender of patients, their geographical location, and the morphology of their root and canal structures. The level of significance was established at 5% (p < 0.05).

## Results

**Figure 1:**
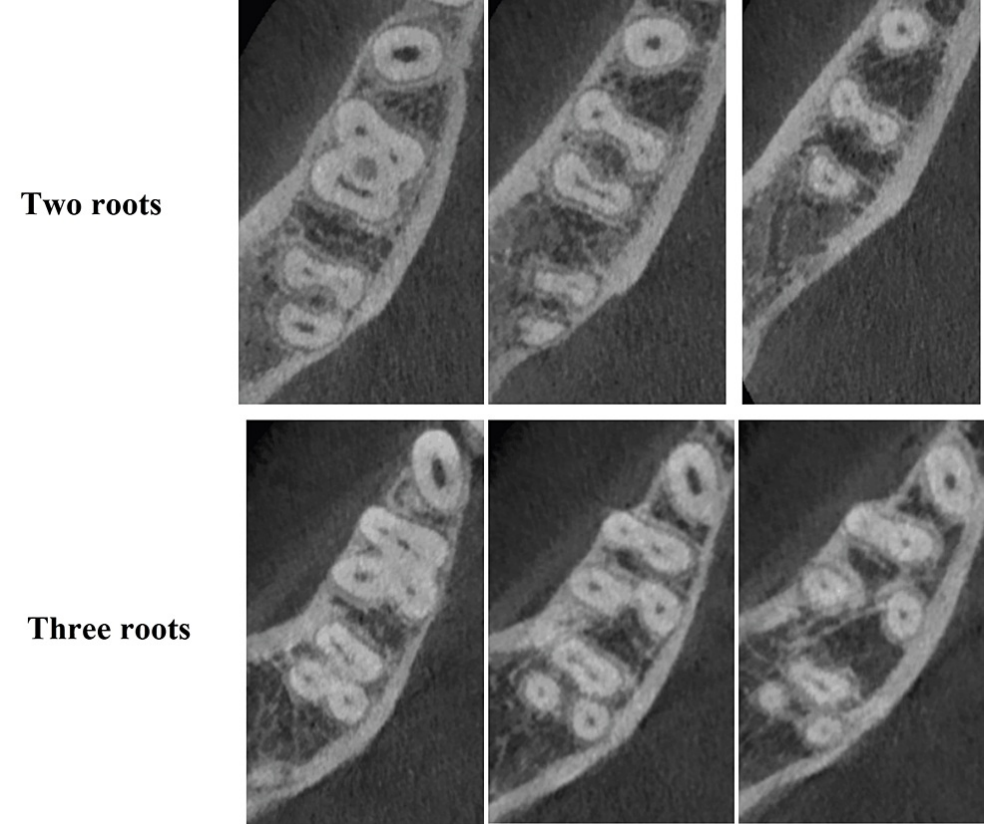
Mandibular first molar with two and three roots

Table 1 displays the distribution of the number of roots observed in the mandibular first and second molars within the sample under investigation. In the case of mandibular first molars, it was observed that 95.3% of them exhibited a bifurcated root structure, while the remaining 4.7% displayed a trifurcated root configuration within the sample population under investigation (Figure 1).

**Table 1:** Number of roots in the first and second mandibular molars

Type of molar		N (%)
Mandibular first molars	One root	0 (0)
	Two roots	486 (95.3)
	Three roots	24 (4.7)
Mandibular second molars	One root	3 (0.6)
	Two roots	494 (96.9)
	Three roots	13 (2.5)

**Figure 2:**
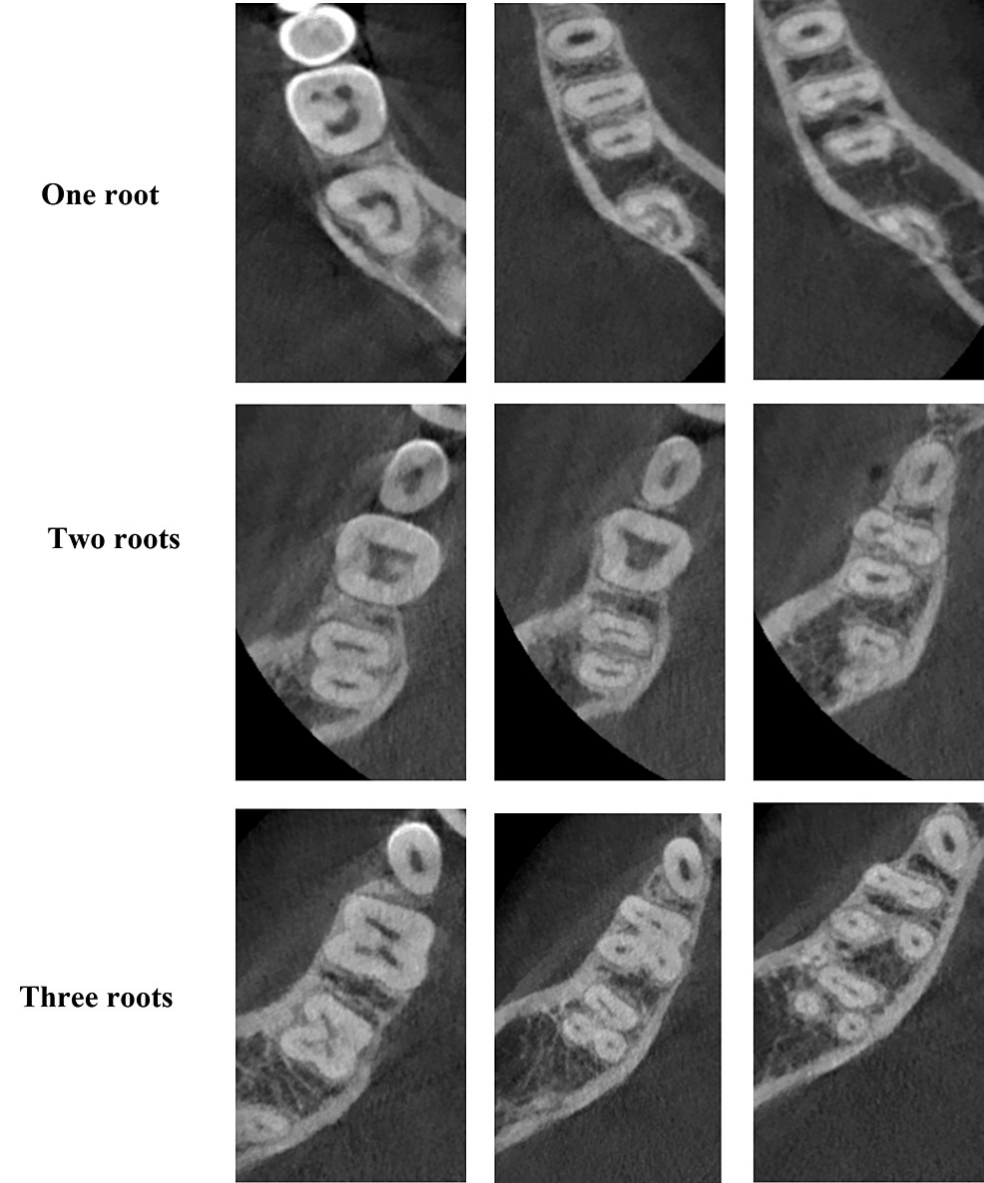
Mandibular second molar with one, two, and three roots

In the case of mandibular second molars, it was observed that 96.9% of them exhibited a bifurcated root structure, while only 2.5% displayed a trifurcated root configuration, and a mere 0.6% possessed a single root (Figure 2).

Table 2 displays the respective quantities of roots found in the mandibular first molar for both males and females. In terms of comparison, it was observed that 45.7% of the research sample consisted of males with two roots, whereas the corresponding figure for females in the study sample was 49.6%. In the study, it was shown that females had a higher likelihood of possessing three roots compared to males (2.9% and 1.8%, respectively). Although there were some variations, no significant differences in the number of roots were observed between males and females (p > 0.05). Additionally, it was observed that there was no statistically significant variation in the quantity of roots observed between the left and right sides (p > 0.05).

**Table 2:** Number of roots for gender and tooth position of mandibular first molars

Number of roots	Gender			Tooth position		
	Male	Female	Total	Left side	Right side	Total
One root n (%)	0 (0)	0 (0)	0 (0)	0 (0)	0 (0)	0 (0)
Two roots n (%)	233 (45.7)	253 (49.6)	486 (95.3)	243 (47.6)	243 (47.6)	486 (95.3)
Three roots n (%)	9 (1.8)	15 (2.9)	24 (4.7)	12 (2.4)	12 (2.4)	24 (4.7)
Total	242 (47.5)	268 (52.5)	510 (100)	255 (50)	255 (50)	510 (100)
p-value	p = 0.103			p = 0.165		

Table 3 presents the data pertaining to the number of roots found in the second mandibular molars among individuals of both genders. The study sample consisted of 50.2% females, whereas 46.7% of the research sample comprised two male individuals. The prevalence of three roots was found to be higher in females compared to males, with rates of 1.8% and 0.8%, respectively. There was a little, albeit not statistically significant, variation in the number of roots between males and females (p > 0.05). Furthermore, it was observed that there was no statistically significant difference in the number of roots between the left and right sides (p > 0.05).

**Table 3:** Number of roots for gender and tooth position of mandibular second molars

Number of roots	Gender			Tooth position		
	Male	Female	Total	Left side	Right side	Total
One root n (%)	0 (0)	3 (0.6)	3 (0.6)	1 (0.2)	2 (0.4)	3 (0.6)
Two roots n (%)	238 (46.7)	256 (50.2)	494 (96.9)	247 (48.4)	247 (48.4)	494 (96.9)
Three roots n (%)	4 (0.8)	9 (1.8)	13 (2.5)	7 (1.4)	6 (1.2)	13 (2.5)
Total	242 (47.5)	268 (52.5)	510 (100)	255 (50)	255 (50)	510 (100)
p-value	p = 0. 186			p = 0. 175		

## Discussion

The success of endodontic treatment relies on the complete elimination of bacteria from the root canal system and the subsequent prevention of reinfection. Hence, a deficiency in acquiring a thorough understanding of the many complexities associated with the internal structure of teeth could potentially lead to the emergence of infections if a canal is disregarded [24], leading to unfavorable consequences. Historically, the management of lower molars has presented a significant problem due to their distinct anatomical characteristics [25].

A variety of approaches have been utilized in the investigation of the quantity of canals inside dental clusters. Two methodologies that provide accurate and detailed information include computerized microtomography and the diaphanization process. Nevertheless, it is crucial to acknowledge that these approaches are restricted to the evaluation of extracted teeth [26]. The acknowledgment of the limitations of two-dimensional radiography techniques for diagnostic purposes and the difficulties in standardizing intricate root canal architecture have been documented [27]. Furthermore, it is important to acknowledge that the precision and reliability of data derived from in vitro studies on dental specimens may be influenced by several factors, such as limits in the modeling process and dental conditions such as pathologies and resorptions [19].

The utilization of CBCT has become increasingly prominent in the field of dentistry as a result of technological improvements. This imaging modality facilitates a thorough examination of dental structures, encompassing the assessment of tooth morphology, analysis of root canal morphology and changes, and exploration of general dental well-being. Cone-beam computed tomography is commonly utilized in research related to the morphological analysis of the root canal system in the field of endodontics. Clinicians must possess comprehensive knowledge about the prevalence, anatomical location, and variability in root canal designs of root-end resections to optimize the effectiveness of endodontic therapies. According to the American Association of Endodontists, the utilization of CBCT is advocated for the aim of discerning anatomical variations and investigating challenging clinical scenarios [28]. According to previous research, CBCT has demonstrated enhanced diagnostic capacities in comparison to periapical radiography for the detection and localization of root canals [24]. The utilization of this noninvasive imaging technique facilitates the capture of three-dimensional pictures, encompassing axial, sagittal, and coronal planes, hence augmenting the precision of diagnosis in healthy teeth.

The objective of this study was to investigate the frequency of DLRs in mandibular molars using CBCT images. A dental practitioner should use caution when doing RCT on the permanent mandibular molars due to the presence of a dental loop. The identification of an additional root can be helped by visually inspecting the clinical crown since it is sometimes associated with an elevated count of cusps, root canals, or lobes [29]. The comprehensive evaluation of diagnostic radiographs plays a pivotal role in the strategic development of an endodontic treatment strategy [29]. According to existing literature, it is advisable to acquire and assess radiographs from several angles [30]. When the radiographic representation of the root canal outlines of the distal root is unclear, it is recommended to take into account the potential presence of a hidden third root. To substantiate this discovery, several academic publications suggest obtaining a second radiograph at a mesial or distal angulation of 30 degrees. Previous studies have demonstrated that this methodology is effective in facilitating the identification of a radix [31].

The mandibular first molars are the first permanent teeth that emerge within the oral cavity. Moreover, it is worth noting that these teeth represent permanent dentition, which is particularly susceptible to dental caries and frequently requires endodontic intervention throughout their functional duration in the oral cavity. An accurate diagnosis of REs is essential in order to optimize the effectiveness of endodontic treatment on these teeth and minimize the risk of recurring apical infections [19]. Previous studies have reported that radicular extensions are frequently detected in the first molars of the mandible [5,32,33].

The existing body of research encompasses documented data pertaining to anatomical changes observed in mandibular molars. Nevertheless, a considerable percentage of general practitioners lack a full comprehension of the varied anatomical configurations of the root canal system in molars [34]. The degree of diversity in the shape of root canals within the distal root of mandibular molars may not be well acknowledged within the overall body of information [35]. The presence of RE has been reported in mandibular molar teeth, namely in close proximity to the second DLC. The prevalence of RE varies among different groups, ranging from 0.9% to 40%. Research [36] indicated a notable disparity in the occurrence of root elongation in mandibular first molars between the Middle Eastern and Eastern Asian populations, with the former exhibiting significantly lower rates. Based on the findings of Zaatar et al. [37], a research investigation conducted on the demographics of Kuwait revealed that a total of 2.7% (four out of 147) of the subjects exhibited mandibular molars characterized by the presence of three roots. Pattanshetti et al. [38] subsequently reported that a subset of molars displayed a prevalence rate of 4% for the occurrence of three roots. Furthermore, they reported a prevalence rate of 4% in the Jordanian population based on their study of 330 participants, which identified a total of 13 cases. The documented prevalence of the specified range in Saudi Arabia has been seen to fluctuate between 2.3% [39] and 5.97% [37]. The present study's findings reveal a greater incidence rate in comparison to the findings reported by Younes et al. [40] while contrasting with the outcomes of Al-Nazhan's study [36]. In our study, the occurrence of radicular enamel was seen in 21% to 50% of mandibular first molars. Radicular cysts may also be detected in the dentition of the second molars; nevertheless, they are primarily encountered in the mandibular first molars. According to the meta-analysis conducted by Martins et al. [22], the stated global prevalence rate of RE is 5.6%. In light of Brazil's extensive racial admixture and consequent genetic amalgamation from other continental populations, it is imperative to do further investigations aimed at ascertaining the frequency of anatomical changes, specifically pertaining to radix, in the first and second lower molars. Although the prevalence of radix in the Brazilian population is currently low, additional research is required to comprehensively ascertain the scope of these abnormalities.

The therapeutic significance of this study is derived from its discovery that the effectiveness of endodontic therapy stays unaltered, even when dental practitioners confront difficulties in accurately recognizing all root canals. The available scholarly sources and the findings of this study collectively suggest a significant difference in the occurrence of root end curvature between East Asian and Western populations. Specifically, there is a higher prevalence of RE in mandibular first permanent molars among individuals of East Asian heritage. The discrepancies identified in the present study when compared to previous research findings, indicate the possible presence of genetically driven variants linked to racial heritage.

Previous studies have demonstrated that the presence of supplementary root structures exhibits variability with respect to gender and symmetry. Nevertheless, the present investigation did not detect any statistically significant alterations in the incidence of these structures when examined based on gender or laterality. The findings of Hatipoğlu et al. [41] and Rodrigues et al. [42] support our research by seeing a higher prevalence in females, while Çolak et al. [43] and Duman et al. [19] documented a larger frequency in males. Nevertheless, Mukhaimer and Azizi [29] found no statistically significant difference between females and males in relation to the prevalence of three-rooted permanent mandibular molars, which is consistent with the results obtained in our investigation.

Regarding the positioning of the radix, many studies, like the research conducted by de Souza-Freitas et al. [44], have indicated a bilateral occurrence of the esthetic region ranging from 50% to 67%. However, Mohan and Thakur [32] did a study that revealed a higher incidence of unilateral radix. Some reports have documented a higher prevalence of enamel renalization on the right side of the mandible, hence presenting conflicting evidence in comparison to the present study, which found no statistically significant gender-based differences in enamel renalization incidence [42-45]. Some studies in the Saudi subpopulation reported that the incidence of RM in the mandibular first molars was between 4.5% and 6.07% [46,47].

Several researchers have illustrated the heightened importance of sophisticated diagnostic tools, such as the dental operating microscope and CBCT, in contrast to conventional diagnostic techniques. Cone-beam computed tomography enables clinicians to accurately visualize the morphology of root canals, assess the number of canals, and establish the spatial orientation of any additional roots [45]. During the process of performing root-end resections, various problems may be encountered as a result of architectural design variables. These factors encompass furcal or strip perforation, root weakening, vertical root fracture, root canal widening, transportation, reduction in study length, and tool fractures. The endodontic access chamber in the first and second mandibular molars is frequently distinguished by its rectangular morphology.

A limitation of the current study pertains to the constrained scope of data collection, as it was exclusively carried out at a singular establishment located in Ha'il, Saudi Arabia. Furthermore, the study utilized a solitary periapical radiograph that was horizontally angled in two dimensions. Cone beam computed tomography efficiently overcomes these constraints by minimizing the problem of superimposition and facilitating improved viewing of three-dimensional anatomical structures. The discrepancies identified between the results of the present study and prior studies may be attributed to differences in ethnicity, race, and research methods. Although the presence of RE and paramolaris was not commonly observed in the study population, accurately identifying the radix is essential for ensuring successful endodontic treatment. The improvement of identification for this unique aberration necessitates the implementation of enhanced investigation techniques and detection methodologies. 

## Conclusions

In light of the limitations inherent in the present investigation, it can be concluded that the discovered RE roots were predominantly found in the mandibular first molar, displaying a tendency for bilateral occurrence in both males and females. The observed frequency of RE among the examined sample was comparatively low. The determination of the radix is crucial for the attainment of an effective endodontic intervention. In order to accomplish enhanced identification of this specific anomaly, it is important to employ advanced investigation approaches and detection methodologies.
